# Alterations in brain networks in children with sub-threshold autism spectrum disorder: A magnetoencephalography study

**DOI:** 10.3389/fpsyt.2022.959763

**Published:** 2022-08-05

**Authors:** Yuka Shiota, Daiki Soma, Tetsu Hirosawa, Yuko Yoshimura, Sanae Tanaka, Chiaki Hasegawa, Ken Yaoi, Sumie Iwasaki, Masafumi Kameya, Shigeru Yokoyama, Mitsuru Kikuchi

**Affiliations:** ^1^United Graduate School of Child Development, Osaka University, Kanazawa University, Hamamatsu University School of Medicine, Chiba University, and University of Fukui, Kanazawa, Japan; ^2^Japan Society for the Promotion of Science, Tokyo, Japan; ^3^Research Center for Child Mental Development, Kanazawa University, Kanazawa, Japan; ^4^Department of Psychiatry and Neurobiology, Graduate School of Medical Science, Kanazawa University, Kanazawa, Japan; ^5^Institute of Human and Social Sciences, Kanazawa University, Kanazawa, Japan; ^6^Department of Cognitive Science, Macquarie University, Sydney, NSW, Australia

**Keywords:** sub-threshold autism spectrum disorder, magnetoencephalography (MEG), social responsiveness scale (SRS), graph theory, small-worldness

## Abstract

Individuals with sub-threshold autism spectrum disorder (ASD) are those who have social communication difficulties but do not meet the full ASD diagnostic criteria. ASD is associated with an atypical brain network; however, no studies have focused on sub-threshold ASD. Here, we used the graph approach to investigate alterations in the brain networks of children with sub-threshold ASD, independent of a clinical diagnosis. Graph theory is an effective approach for characterizing the properties of complex networks on a large scale. Forty-six children with ASD and 31 typically developing children were divided into three groups (i.e., ASD-Unlikely, ASD-Possible, and ASD-Probable groups) according to their Social Responsiveness Scale scores. We quantified magnetoencephalographic signals using a graph-theoretic index, the phase lag index, for every frequency band. Resultantly, the ASD-Probable group had significantly lower small-worldness (*SW*) in the delta, theta, and beta bands than the ASD-Unlikely group. Notably, the ASD-Possible group exhibited significantly higher *SW* than the ASD-Probable group and significantly lower *SW* than the ASD-Unlikely group in the delta band only. To our knowledge, this was the first report of the atypical brain network associated with sub-threshold ASD. Our findings indicate that magnetoencephalographic signals using graph theory may be useful in detecting sub-threshold ASD.

## Introduction

Autism spectrum disorder (ASD) is a neurodevelopmental disorder characterized by deficits in social cognition and communication and repetitive/restrictive behaviors (1). These autistic traits are distributed on a continuum from the typical developmental range to the diagnostic range (2). As a result, the wide variety of clinical presentations blurs the lines between autistic and typically developing children (3, 4). As one might infer, there are children who do not meet the full diagnostic criteria but still experience difficulties related to autistic traits. Since these problems can still significantly impair an individual’s daily activities, they are referred to as “sub-threshold ASD” (5). Evidence suggests that sub-threshold ASD tends to involve difficulty in reciprocal communication, which could result in maladjustment and increased risk of psychiatric disorders such as depression and anxiety (6). In fact, Moriwaki and Kamio (7) revealed that children with a higher level of autistic traits have a greater risk of additional mental health problems even if they do not meet the full diagnostic criteria of ASD (7). Therefore, early detection and appropriate support for children who have a certain level of autistic traits (i.e., sub-threshold ASD), but do not fulfill the diagnostic criteria of ASD, is necessary to prevent secondary disorders such as depression and anxiety. However, no reliable biological marker currently exists for detecting sub-threshold ASD.

Evidence suggests that ASD is a disorder of “brain connectivity.” In terms of pair-wise brain connectivity, a popular hypothesis is that ASD is characterized by long-range underconnectivity combined with local overconnectivity [in magnetoencephalography (MEG) or electroencephalography (EEG), connectivity usually refers to how electromagnetic signals from different brain regions are similar]. We know that interactions between pairs of regions combine to make a single, large network of the brain. In this context, a novel way to study the brain is to analyze the macroscopic behavior of the brain network as a whole instead of focusing on the respective pair-wise connectivity. Graph theory effectively describes the properties of complex networks on a large scale (8). Using graph theory, we can express geometric features of a given network in terms of numbers referred to as “graph metrics.” Among these, “small-worldness” (*SW*) is considered an index of optimal balance between integrated and segregated information processing in the brain network. *SW* has been well-reported for comparisons of brain networks of children with typical development (TD) and ASD, and these studies have almost consistently reported lower *SW* in children with ASD. Particularly, the brain network of children with ASD shows significantly lower *SW* than that of children with TD in the delta (9), theta combined with alpha (10), and beta (11) bands and every (i.e., delta, theta, alpha, beta, and gamma) frequency band (12) [one exception is a higher *SW* in the gamma band reported by (9)]. It is noteworthy that lower small worldness in the beta band is reported to linearly correlate with severer social impairment in children with ASD (11). Given the relation between *SW* and ASD symptomatology, one might infer that it would be possible to detect children with sub-threshold ASD in early developmental stages using *SW*. However, no graph-theoretical studies have focused on “sub-threshold ASD.”

For sub-threshold ASD, whether one fulfills the diagnostic criteria of ASD would be less meaningful, as the definition of sub-threshold ASD borderlines between ASD and typically developing children. As such, children with a similar degree of difficulty in communication could accidentally be diagnosed or not with ASD. In this study, we recruited children, with ASD and TD and classified them into three groups (ASD-Unlikely, ASD-Possible, and ASD-Probable) in terms of difficulties both in communication and repetitive restricted behaviors, rather than them being formally diagnosed with ASD. We combined MEG and graph theory to investigate the brain network of these three groups. We hypothesized that children in the ASD-Possible group would have atypical brain networks differing from those in the ASD-Unlikely or ASD-Probable group. Specifically, we hypothesized that there may be differences in *SW* in relation to the degree of autistic traits.

## Materials and methods

### Participants

We enrolled 54 Japanese children with ASD (36 boys, 18 girls, aged 38–92 months) and 31 children with TD (26 boys, 5 girls, aged 53–89 months) from the Kanazawa University hospital and affiliated hospitals. ASD was diagnosed following the Diagnostic and Statistical Manual of Mental Disorders (DSM) IV (1) using the Diagnostic Interview for Social and Communication Disorders (13) or the Autism Diagnostic Observation Schedule-Generic (14) and the Autism Diagnostic Observation Schedule 2 (15). We excluded participants with blindness, deafness, other neuropsychiatric disorders, and those receiving an ongoing medication regimen. We assessed the intelligence of the participants using the Japanese version of the Kaufman Assessment Battery for Children (K-ABC) (16) and excluded eight participants who scored <70 on the Mental Processing Scale. Consequently, 46 children with ASD (31 boys, 15 girls, aged 38–92 months) and 31 with TD (26 boys, 5 girls, aged 53–89 months) remained for analysis. The Social Responsiveness Scale (SRS) (17) was used to assess the participants’ autistic traits (a detailed explanation of the SRS is available in the Supplementary Data). We separated the participants into three groups in terms of the T-score of the SRS according to a previous study (18); (i) ASD-Unlikely group (children in the normal range, T-scores less than 59), (ii) ASD-Possible group (children with less obvious autistic traits corresponding to sub-threshold ASD, T-scores between 60 and 75), and (iii) ASD-Probable group (children with obvious autistic traits, T-scores greater than 76). The study was approved by the Ethics Committee of Kanazawa University Hospital and conducted following the Declaration of Helsinki. Written informed consent was obtained from all parents.

### Magnetoencephalography recordings

Magnetoencephalography data were recorded using a 151-channel Superconducting Quantum Interference Device whole-head coaxial gradiometer MEG system for children (PQ 1151 R; Yokogawa/KIT, Kanazawa, Japan) in a magnetically shielded room (Daido Steel, Nagoya, Japan) installed at the MEG Center of Ricoh Company, Ltd. (Kanazawa, Japan). The band-pass-filtered MEG data (0.16–200 Hz) were collected at a sampling rate of 2,000 Hz. We ensured that each child was motionless during the recording (a detailed description of the recording procedure is available in the Supplementary Data).

### Co-registration of magnetoencephalography on substituted or participants magnetic resonance image

A total of 25 participants were unable to remain motionless during the magnetic resonance image (MRI) recordings. We employed a suitable MRI brain template for each such participant based on the individual head surface shapes using an algorithm that we had developed for previous studies (see details in Supplementary Data). To use a brain template, we attached three coils at each bilateral mastoid process and the nasion. The other 21 children underwent complete MRI. For those, to use their own MRI image, we attached four coils at each of the bilateral mastoid processes, midline frontal, and vertex. In either case, in reference to the specific magnetic field generated by the coils, we could ascertain the child’s head position within the helmet. A detailed description of the procedure is available in Supplementary Data.

### Co-registration of magnetoencephalography on participant’s own magnetic resonance image image

A newly developed silent mode MRI and a sequence allowed us to capture the participants’ brain structure in less than 30 s (19). Consequently, with the help of an MRI-compatible video presentation system and an MRI-compatible headphone system, we were able to conduct MRI without sedation for the other 21 children. The following methods for MR image acquisition have been described before (11). A 1.5-T MRI scanner (SIGNA Explorer; GE Healthcare, United States) was used to collect structural brain images from all participants. The three-dimensional high-resolution T1-weighted gradient-echo and Silenz pulse sequence images were used as an anatomical reference. The imaging parameters were as follows: TR = 435.68 ms, TE = 0.024 ms, flip angle = 7°, FOV = 220 mm, matrix size = 256 × 256 pixels, slice thickness = 1.7 mm, total of 130 transaxial images.

For these 21 participants, we co-registered the MEG and their own MR images following the marker locations. The markers for MEG and MRI were the frontal midline, parietal, and bilateral mastoid processes. For the MEG, we used four coils to generate a magnetic field. For MRI, we used four pieces of lipid capsule as markers. Furthermore, we identified points on the mastoid processes, nasion, and skull surface visually on MRI. Approximately 15–25 points were depicted for each participant.

### Magnetoencephalography data analyses

The MEG analyses were performed using Brainstorm.^1^ We preprocessed the MEG data according to recommendations of the Organization for Human Brain Mapping (20). Then, we estimated the brain signal sources using an anatomically constrained MEG approach. The signal sources were grouped into 68 regions represented in the Desikan–Killiany atlas using principal component analyses. The data were segmented into 5-s epochs; then, each epoch was band-pass filtered for the used frequency bands: delta (2–4 Hz), theta (4–8 Hz), alpha (8–13 Hz), beta (13–30 Hz), and gamma (30–60 Hz). This procedure was identical to those used in our earlier studies (11, 21). The details regarding the procedure (i.e., preprocessing, atlas-guided source reconstruction, and segmenting) are available in the Supplementary Data.

The phase lag index (PLI) was used to measure the functional connectivity between brain regions (a detailed explanation is available in Supplementary Data). We chose the PLI as it is insensitive to the field spread and has been widely used to describe atypical brain networks in children with ASD (9–12).

### Graph construction and graph metrics

The basic topography of a network is represented by a graph consisting of “nodes” and “edges” connecting pairs of nodes. In this study, nodes corresponded to 68 brain regions of the Desikan–Killiany atlas (22). We weighted the edges based on the PLI values between pairs of brain regions. For each epoch, we constructed an undirected weighted functional connectivity matrix (68 × 68) based on the PLI for each frequency band (i.e., delta, theta, alpha, beta, and gamma). We averaged the matrices of all epochs for each participant. For those averaged matrices, weak connectivity may represent spurious connections. To remove possible spurious connections, we binarized those graphs through the application of a proportional weight threshold. We set proportional threshold κ, the proportion of total connections retained, as 0.2 (a κ of 0.2 indicates that the strongest 20% of the connections were selected) in accordance with earlier studies (6, 8). At present, however, there is no formal consensus regarding the selection of a weight threshold. To verify the stability of the results, we also investigated a range of proportional thresholds κ from 0.1 to 0.3 at 0.02 increments.

In this manner, a complex brain network is expressed as graphs in terms of binary matrices. The information included in those graphs can be further summarized *via* various measures. Well-established measures include the mean clustering coefficient (*C*), average shortest path length (*L*), and *SW*. *C* represents how clustered a graph’s nodes are and is a commonly used measure of functional segregation. *L* is the average shortest path length between all node pairs in the network and is the most commonly used measure of functional integration. Notably, human brain networks have higher *C* and shorter *L*, which purportedly represents an optimal balance between integration and segregation. Such a network is called a small-world network. In this context, *SW*, another graph metric, is the ratio of normalized *C* and normalized *L*. As such, a graph with a high *SW* is a network significantly more clustered than a random network yet has approximately the same characteristic path length (a detailed explanation is available in Supplementary Data). In this study, we calculated each participant’s *SW, C, and L* on each frequency band. In addition to these network-level properties, we also investigated node-level properties to locate regions with atypical properties. In particular, the clustering coefficient and average shortest path length were calculated at each node.

### Statistical analysis

All statistical analyses were performed using Stata (ver. 16.1; Stata Corp., College Station, TX, United States). We used Student’s *t*-test to compare differences in age and intelligence quotient (IQ: the Mental Processing Scale scores in the K-ABC) between children with ASD and those with TD. Sex differences were examined *via* a chi-square test.

We used analysis of variance to compare the three groups (ASD-Unlikely, ASD-Possible, and ASD-Probable groups) in terms of age, sex, scores in the Mental Processing Scale and the Achievement Scale of the K-ABC, and SRS total T-score).

Subsequently, to test the differences in network-level graph metrics (*C*, *L*, and *SW*) among the three groups, we fitted a generalized linear model with a gamma error distribution with a log link (i.e., a gamma regression model). Particularly, we predicted log-transformed graph metrics based on the groups (treated as a categorical variable), age, sex, and Mental Processing Scale of the K-ABC. As the test was repeated three times for each frequency band (ASD-Unlikely vs. ASD-Probable, ASD-Unlikely vs. ASD-Possible, ASD-Probable vs. ASD-Possible), adjusted *p*-values were obtained using a false discovery rate (FDR) approach (23) for three comparisons, and significance was inferred for adjusted *p*-values of <0.05.

To test the differences in node-level graph metrics (*C* and *L*) among the three groups, we similarly predicted log-transformed graph metrics based on the groups, age, sex, and Mental Processing Scale of the K-ABC. As the test was repeated 3 (ASD-Unlikely vs. ASD-Probable, ASD-Unlikely vs. ASD-Possible, ASD-Probable vs. ASD-Possible) * 68 (the number of nodes) = 204 times for each frequency band, FDR-adjusted *p*-values (23) were calculated for 204 comparisons, and significance was inferred for adjusted *p*-values of <0.05.

As our primary focus was *SW*, as exploratory analysis, we investigated linear relations between *SW* in each frequency band and the SRS scores. In particular, we predicted *SW* metrics based on the SRS total T-scores.

## Results

There were no significant differences in ages, sexes, and K-ABC scores between children with ASD and TD. The participant and group characteristics are shown in Tables 1, 2.

**TABLE 1:** Participants’ characteristics.

	ASD *N* = 46	TD *N* = 31	χ^2^ or *t*	*p*
Age in months	66.3 (12.00)	69.2 (9.73)	1.13	0.26
Sex (% Male)	67.3%	83.9%	2.62	0.11
K-ABC mental processing scale	102.9 (15.30)	107.8 (12.62)	1.49	0.14
K-ABC achievement scale	100.4 (16.89)	103.0 (14.30)	0.71	0.48
SRS total T-score	71.9 (10.46)	50.7 (7.76)	−9.60	0.00**

Numbers are mean (standard deviation).

**represents a significant difference (p < 0.01).

ASD, autism spectrum disorder; K-ABC, Kaufman Assessment Battery for Children; SRS, Social Responsiveness Scale; TD, typical development.

**TABLE 2:** Group characteristics.

	ASD-Unlikely *N* = 31	ASD-Possible *N* = 29	ASD-Probable *N* = 17	*F*	*p*
Age in months	69.4 (10.27)	66.2 (10.39)	65.9 (13.90)	0.81	0.45
Sex (% Male)	80.6%	75.9%	64.7%	0.74	0.48
K-ABC mental processing scale	109.1 (12.87)	101.8 (15.57)	102.5 (13.91)	2.27	0.11
K-ABC achievement scale	104.0 (14.19)	97.1 (14.00)	104.2 (20.46)	1.77	0.18
SRS total T-score	49.3 (5.97)	67.0 (4.52)	82.8 (5.19)	230.57	0.00**

Numbers are mean (standard deviation).

**Represents a significant difference (p < 0.01).

ASD, autism spectrum disorder; K-ABC, Kaufman Assessment Battery for Children; SRS, Social Responsiveness Scale; TD, typical development.

### Group differences in network-level graph metrics

We first investigated the network-level properties in each frequency band setting κ at 0.2. Hereafter, adjusted *p*-values are presented because statistical significance was inferred based on the adjusted *p*-values.

**FIGURE 1:**
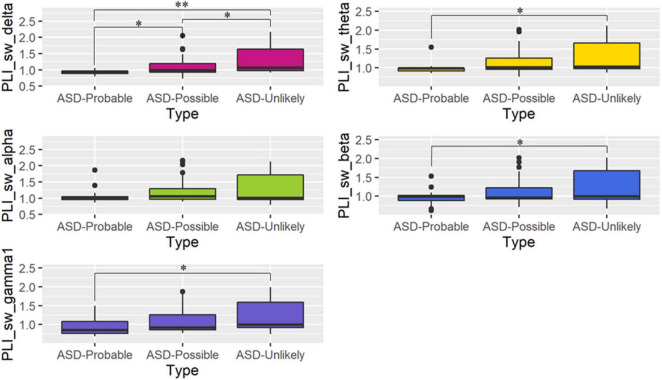
Boxplot of small-worldness (*SW*) in each frequency band. The panels present the phase lag index values of *SW* among the three groups for each frequency band (mean ± SD). *Post-hoc* comparisons between the autism spectrum disorder (ASD)-Probable and ASD-Unlikely, ASD-Probable and ASD-Possible, and ASD-Possible and ASD-Unlikely groups: *p < 0.05, ^**^p < 0.000.

Gamma regression models revealed that the ASD-Probable group had significantly lower *SW* than the ASD-Unlikely group in all frequency bands except for the alpha band. For the delta band, significant differences were observed in all three comparisons. In particular, the ASD-Probable group had significantly lower *SW* in the delta band than the ASD-Unlikely group (coefficient = −0.32, 95%CI: −0.49, −0.16, *p* = 0.0001); they also had a significantly lower *SW* than the ASD-Possible group (coefficient = −0.18, 95%CI: −0.34, −0.01, *p* = 0.044). In addition, the ASD-Possible group had a significantly lower *SW* in the delta band than the ASD-Unlikely group (coefficient = −0.15, 95%CI: −0.29, −0.00, *p* = 0.044). For the theta band, the ASD-Probable group had significantly lower *SW* than the ASD-Unlikely group (coefficient = −0.26, 95%CI: −0.43, −0.08, *p* = 0.011). For the beta band, the ASD-Probable group had significantly lower *SW* than the ASD-Unlikely group (coefficient = −0.25, 95%CI: −0.45, −0.06, *p* = 0.031). For the gamma band, the ASD-Probable group had an almost significantly lower *SW* than the ASD-Unlikely group (coefficient = −0.23, 95%CI: −0.41, −0.04, *p* = 0.05). On the other hand, there were no significant differences in *C* and *L* based on the groups. Only models predicting *SW* had a significant main effect on group. Figure 1 and Table 3 summarize the differences in graph metrics among the groups. FDR-corrected *p*-values are presented in Table 3. The effect of other variables (i.e., age, sex, and scores on the Mental Processing scale of the K-ABC) are provided in Supplementary Table 1.

**TABLE 3:** Differences in graph metrics.

Comparison	Frequency band	Coefficient	*95% CI*			*p*
	Delta					
ASD-Unlikely vs. ASD-Possible	*C* *L* *SW*	−0.044 0.015 −0.146	−0.248 −0.037 −0.289	–	0.160 0.068 −0.004	0.675 0.781 0.044*
ASD-Unlikely vs. ASD-Probable	*C* *L* *SW*	−0.271 −0.009 −0.322	−0.508 −0.069 −0.485	–	−0.033 0.052 −0.158	0.077 0.781 0.000**
ASD-Possible vs. ASD-Probable	*C* *L* *SW*	−0.227 −0.024 −0.175	−0.460 −0.084 −0.337	–	0.005 0.036 −0.014	0.083 0.781 0.044*
	**Theta**					
ASD-Unlikely vs. ASD-Possible	*C* *L* *SW*	−0.036 0.000 −0.085	−0.227 −0.047 −0.236	–	0.155 0.048 0.066	0.715 0.984 0.270
ASD-Unlikely vs. ASD-Probable	*C* *L* *SW*	−0.211 −0.023 −0.257	−0.433 −0.079 −0.430	–	0.010 0.032 −0.083	0.172 0.613 0.011*
ASD-Possible vs. ASD-Probable	*C* *L* *SW*	−0.176 −0.024 −0.172	−0.394 −0.079 −0.342	–	0.043 0.031 −0.001	0.172 0.613 0.072
	**Alpha**					
ASD-Unlikely vs. ASD-Possible	*C* *L* *SW*	0.022 −0.001 −0.039	−0.144 −0.043 −0.206	–	0.188 0.041 0.128	0.798 0.968 0.648
ASD-Unlikely vs. ASD-Probable	*C* *L* *SW*	−0.135 −0.022 −0.184	−0.327 −0.071 −0.376	–	0.058 0.027 0.009	0.256 0.597 0.184
ASD-Possible vs. ASD-Probable	*C* *L* *SW*	−0.156 −0.021 −0.145	−0.346 −0.069 −0.333	–	0.033 0.027 0.044	0.256 0.597 0.198
	**Beta**					
ASD-Unlikely vs. ASD-Possible	*C* *L* *SW*	−0.010 0.005 −0.099	−0.199 −0.034 −0.267	–	0.178 0.045 0.070	0.914 0.785 0.251
ASD-Unlikely vs. ASD-Probable	*C* *L* *SW*	−0.152 −0.012 −0.254	−0.371 −0.058 −0.448	–	0.067 0.034 −0.060	0.297 0.785 0.031*
ASD-Possible vs. ASD-Probable	*C* *L* *SW*	−0.141 −0.018 −0.156	−0.356 −0.063 −0.346	–	0.073 0.028 0.036	0.297 0.785 0.167
	**Gamma**					
ASD-Unlikely vs. ASD-Possible	*C* *L* *SW*	−0.026 0.008 −0.083	−0.202 −0.033 −0.243	–	0.150 0.049 0.077	0.771 0.704 0.310
ASD-Unlikely vs. ASD-Probable	*C* *L* *SW*	−0.094 −0.031 −0.227	−0.300 −0.078 −0.412	–	0.114 0.016 −0.041	0.762 0.294 0.0498*
ASD-Possible vs. ASD-Probable	*C* *L* *SW*	−0.068 −0.040 −0.144	−0.270 −0.086 −0.325	–	0.134 0.008 0.038	0.762 0.294 0.182

All p-values are false discovery rate corrected.

*represents a significant difference (p < 0.05), **represents a significant difference (p < 0.01).

ASD, autism spectrum disorder; C, mean clustering coefficient; L, average shortest path length; SW, small-worldness.

Similar patterns were observed for the other κ values. The ASD-Unlikely group showed higher *SW* than the ASD-Probable group, and the values for the ASD-Possible group lied somewhere in between. The differences in *SW* between the ASD-Unlikely and ASD-Probable groups were significant for the delta, theta, and beta bands (the differences in the theta and delta bands were significant for every κ from 0.10 to 0.30, and the differences in the beta band were significant when κ was set at 0.10–0.22). Supplementary Figure 1 and Supplementary Table 2 present the relevant results.

### Group differences in node-level graph metrics

We first investigated the node-level properties in each frequency band setting κ at 0.2. After multiple-testing correction, node-level *C* was significantly different in the nodes corresponding to the right fusiform, right superior parietal (in the alpha band), right supramarginal (in the theta band), and right transverse temporal (in the beta band) areas. These node-level significant differences were observed in the comparison between ASD-Possible and ASD-Probable individuals, except for the right superior parietal where the difference was significant between the ASD-Unlikely and ASD-Probable groups. These results highlight that the difference between the ASD-Unlikely and ASD-Possible groups could not have been detectable if we had only focused on the node-level properties.

Afterward, we investigated the node-level properties for the other κ values. Among the above regions, only the difference in the right transverse temporal (in the beta band) remained significant for the other κ values (when κ was set at 0.22–0.26). These results indicate that the difference in node-level properties *per se* could not explain the observed difference in network-level properties. Supplementary Table 3 presents the relevant results.

### Linear relation between small-worldness and social responsiveness scale total T-scores

Subsequently, we investigated the relationship of *SW* with the SRS total T-scores using linear regression models. The regression models showed that lower *SW* in the delta, theta, beta, and gamma bands was significantly associated with higher SRS total T-scores (*t*_(75)_ = −4.02, *p* = 0.0001, *t*_(75)_ = −3.04, *p* = 0.003, *t*_(75)_ = −2.57, *p* = 0.012, *t*_(75)_ = −2.50, *p* = 0.015, respectively). The relevant results are presented in Supplementary Figure 2 and Supplementary Table 4.

## Discussion

To our knowledge, this is the first graph-theoretical study focusing on “sub-threshold ASD.” Our results revealed that each group—ASD-Unlikely, ASD-Possible, and ASD-Probable—as determined based on the SRS, has peculiar brain networks in terms of *SW*, a measure of optimal balance between functional integration and segregation. Particularly, the ASD-Probable group showed significantly lower *SW* than the ASD-Unlikely group in the delta, theta, and beta bands. These results suggest that the brain network of ASD-Probable individuals deviates from an unaffected brain’s small-world properties, and the brain network of ASD-Possible individuals falls somewhere in between those of the ASD-Probable and the ASD-Unlikely groups. The differences between the ASD-Possible group and the other two groups were significant even in the delta band.

Among the previous graph-theoretical studies of children with ASD, lower *SW* was almost consistently reported in various frequency bands [delta (9), theta combined with alpha (10), beta (11), and all frequency bands (12)]. We used the same connectivity measure (i.e., the PLI) as those studies (9–12) and confirmed the lower *SW* in ASD-Probable individuals. Considering the substantial methodological differences, such as the varying properties of the graphs [weighted (10, 12) vs. binary graphs (9, 11)], electromagnetic field measures [EEG (10, 12) vs. MEG (9, 11)], analytic spaces [sensor space (9, 10, 12) vs. source space (11)], and recording conditions [e.g., the presence of visual stimulation (9, 10), fixation cross (11), or natural environment (12)], it is surprising that lower *SW* is almost consistently reported. Notably, studies with relatively small sample sizes [i.e., the number of children with ASD vs. those with TD was 24 vs. 24 in (9), 12 vs. 19 in (10), and 20 vs. 25 in (11)] have reported inconsistent results across the frequency bands, whereas studies with larger sample sizes [80 vs. 106 in (12) and 46 vs. 31 in this study] have reported lower *SWs* for ASD/ASD-Probable children across the frequency bands. Combining the existing evidence, the ASD brain may be considered to exhibit lower *SW* in every frequency band, but the difference is not as large as those reported by studies with small sample sizes. In this context, our study’s results reinforce the notion that there is atypical information processing in the autistic brain and further support the usefulness and robustness of graph theory to describe the differences in network structures. In particular, using a combination of MEG/EEG and the graph theoretical approach, one would be able to effectively detect a characteristic of the ASD brain (e.g., lower *SW* in the delta band) regardless of some methodological differences (e.g., measures for the electromagnetic field and recording conditions).

This study was the first to show that an atypical brain network (i.e., lower *SW* in the delta band) is detectable even in individuals with less evident autistic traits (i.e., ASD-Possible individuals). It is noteworthy that the difference in node-level properties could not by itself explain this network-level difference. More importantly, we could not find a significant difference between the ASD-Possible and ASD-Unlikely groups in terms of node-level properties. These findings suggest that (i) the less evident autistic traits might not arise from atypical local information processing and may rather reflect atypical information processing at the network level and (ii) node-level properties might be insufficient to distinguish individuals with less evident autistic traits from the general population. In this sense, this study highlights the possible usefulness of graph theory for the detection of individuals with sub-threshold ASD, which could not be achieved using traditional approaches such as area by area comparisons. This particular application may be attractive from a clinical perspective because sub-threshold ASD is often overlooked, although the affected individuals tend to experience difficulties in social adaptation, and thus need support, as do children who meet the diagnostic criteria for ASD.

Finally, in the exploratory analysis of the linear relation between *SW* and the SRS scores, we found significant associations between these two metrics in all frequency bands except alpha. In this context, we reconfirmed the previously reported association for the beta band (11) and extended the previous findings in that the relation between *SW* and autistic symptoms could be observable in the other frequency bands.

This study had several limitations. First, the sample size was too small. Studies with a larger sample are needed to verify the neural bases of the brain networks in sub-threshold ASD. Second, all participants were young children. It is necessary to include adolescents and adult individuals with sub-threshold ASD to generalize the results. Third, the children remained motionless in the MEG system with the aid of visual attention (e.g., showing a video program). Because of this, the MEG data were recorded with eyes open under visual stimulation. As a result, the observed brain activity needs to be clearly distinguished from “resting” brain activity. Studies under controlled conditions of attention may provide more reliable evidence; however, these conditions would be difficult to apply to young children.

In conclusion, our results revealed varying atypical neural network properties according to the degree of autistic traits. Our findings highlight a theory of altered brain connectivity in ASD, which focuses on atypical *SW* network properties assessed using graph theory. Our results indicate that *SW* derived from graph theory, when applied to a MEG signal, may help characterize the neural basis of sub-threshold ASD.

## Data availability statement

The raw data supporting the conclusions of this article will be made available by the authors, without undue reservation.

## Ethics statement

This study was approved by the Ethics Committee of Kanazawa University Hospital and conducted following the Declaration of Helsinki. Written informed consent was obtained from the minor(s)’ legal guardian/next of kin for the publication of any potentially identifiable images or data included in this article.

## Author contributions

YS: conceptualization and writing – original draft. YS and TH: methodology and project administration. YS, DS, TH, and MaK: software. YS, DS, and TH: formal analysis and data curation. YY, ST, CH, and KY: investigation. YY, ST, CH, KY, and SI: resources. YS, TH, and MiK: funding acquisition. MiK and SY: supervision. DS, TH, and MiK: writing – review and editing. All authors contributed to the article and approved the submitted version.

## Footnotes

1
https://neuroimage.usc.edu/brainstorm/Introduction
