# Supplementary material for: Alterations in brain networks in children with sub-threshold autism spectrum disorder: A magnetoencephalography study
Source: Front Psychiatry. 2022 Aug 5;13:959763. doi: 10.3389/fpsyt.2022.959763 (PMC9390481; doi:10.3389/fpsyt.2022.959763)
Supplement: Supplementary file 1 [file Data_Sheet_1.docx]

***Supplementary Material***

1 Supplementary Data

**1.1 Assessment of autistic traits - Social Responsiveness Scale**

The Social Responsiveness Scale (SRS) (1), a 65-item rating scale that measures social impairment and autistic mannerisms, was used to assess the participants’ autistic traits. We used the sex-normed T scores (SRS-T) of five subscales: social awareness, social cognition, social communication, social motivation, and autistic mannerisms. Higher scores represent greater autistic traits. This parent-rating questionnaire has been commonly used as a quantitative measure of autistic traits and has robust measurement properties in the general population and clinical cases of ASD (2).

To investigate whether the graph metrics of the functional brain networks differed depending on the degree of autistic traits, we categorized the participants into three groups in terms of the T-scores of the SRS, according to a previous study (3); (i) children within the normal range (ASD-Unlikely group, T-score ≤ 59), (ii) children with an increased number of autistic traits almost corresponding to subthreshold ASD (ASD-Possible group, T-score ≥ 60 and ≤ 75), and (iii) children with the greatest autistic traits corresponding to the threshold level (ASD-Probable group, T-score ≥ 76).

**1.2 MEG recordings**

A custom-made child-sized MEG device was used to measure brain responses in children. Using the child-sized MEG device makes it easier and more effective to position the sensors and limit head movement (4). Before the recording, we instructed each child not to move their head or body to avoid motion artifacts. We supplied several video programs that were popular among young children so that children could watch their favorite (muted) video programs projected onto a screen during the recording. To further encourage the child to maintain a steady head position, a staff member stayed in the room. In this way, we were able to entertain most of the children. Additionally, they were monitored carefully via video monitoring. If a child’s head position moved from its starting position, those related MEG data were excluded from further analyses.

**1.3 Co-registration of MEG on the substituted magnetic resonance imaging (MRI) template**

A total of 25 participants were unable to remain motionless during the MRI recordings. For those participants, we employed a suitable MRI brain template based on each head surface shape using an algorithm used in previous studies (5,6). Particularly, the MEG and template MR images were co-registered according to the electrical dipole current location, which is generated exactly at the supratemporal auditory cortex in response to auditory syllable sound stimulation. The details of the dipole current estimation and auditory stimuli are described below. To establish a suitable brain template for each child, we prepared a database of MRIs and corresponding head surfaces of Japanese children. Subsequently, we selected a suitable MRI image according to the shape of the head surface for each child. Particularly, (i) we prepared T1-weighted MR images from 98 children (aged 0–8 years), which were considered templates of head surfaces and cortical structure. For each of the 98 template images, five fiduciary points (right preauricular, left preauricular, nasion, vertex, and inion) on the head surface were determined. (ii) We determined each participant’s corresponding five surface points. Subsequently, the distance between the corresponding surface points of the participant and a template was calculated as the root mean square error (RMSE). Particularly, the RMSE was calculated as follows.

$$RMSE=\sqrt{\frac{1}{N}{\sum_{i=1}}^{N}(XC_{i}-XO_{i})^{2}}$$

where *XC_i_* is the child participant coordinates, *XO_i_* is the template coordinates, and *N* denotes the number of surface points (*N* = 5 in our study). (iii) We selected the template with the lowest RMSE among all templates as the optimal brain template for the child.

**1.4 Auditory-evoked field stimuli and procedures**

The following procedures were mostly identical to those used in a previous study conducted in our laboratory (7). To use a brain template, we attached three coils at each of the bilateral mastoid processes and nasion. Typical oddball sequences comprising standard stimuli (456 times, 83%) and deviant stimuli (90 times, 17%) were used. In this stimulus, the Japanese syllable “ne” is pronounced in two different ways. For the standard stimulus, we used a repetitive series of utterances of /ne/ pronounced with a flat tone (/ne/). This stimulus contains no intonational information. As a deviant stimulus, we used “ne” pronounced with a high falling tone (/Ne/). The reason for using this syllable is that “ne” in Japanese is a sentence-final particle and conveys prosodic information (8). Moreover, “ne” is often used in conversations between mothers and children, expressing the speaker’s request for the listener to empathize with them (9). The “ne” sound was pronounced by a female Japanese native speaker and recorded with a condenser microphone (NT1-A; Rode, Silverwater, NSW, Australia). The interstimulus interval (ISI) was 818 ms at a level of approximately 65 dB (A-weighted) against a background noise of 43 dB, on average, as measured using an integrating sound level meter (LY20; Yokogawa, Tokyo, Japan).

**1.5 MEG data analysis**

The MEG analyses were performed using Brainstorm (10), which is documented and freely available for download online under the GNU general public license.

**1.6 Preprocessing**

The MEG data were preprocessed according to recommendations from the Organization for Human Brain Mapping (11). First, we down-sampled the MEG recordings to 500 Hz. Second, noisy sensors were excluded from the analysis. Third, a notch filter was applied to remove the 60-Hz harmonics (60, 120, and 180 Hz), followed by a bandpass filter (0.5–200 Hz). Fourth, the independent component analysis method was used to remove cardiac artifacts and blink artifacts. Fifth, segments containing obvious motion noise were excluded based on visual inspection of the waveforms.

**1.7 Atlas-guided source reconstruction and segmenting**

An anatomically constrained MEG approach that places an anatomical constraint on the estimated sources was used to estimate the brain signal sources (12). When the sources are estimated, each participant's recorded brain activity is assumed to lie in the cortical mantle. A head model was computed using the overlapping spheres algorithm (13) with the default source space (a lower-resolution cortical surface representation with 15,000 vertices). We used weighted minimum-norm estimation to estimate source orientation constraints (14). An identity matrix was used as noise covariance because no noise recording was available. Signal sources were grouped into 68 regions represented in the Desikan–Killiany atlases (15). When we grouped the sources, we used principal component analyses.

The data were segmented into 5-second continuous segments, with a minimum of 10 segments (50-second recording period) allowed per subject. Each epoch was band-pass filtered for the most commonly used frequency bands: delta (2–4 Hz), theta (4–8 Hz), alpha (8–13 Hz), beta (13–30 Hz), and gamma (30–60 Hz). This procedure was identical to those used in our earlier studies (16,17).

**1.8 Phase Lag Index (PLI)**

In the field of electromagnetic brain imaging (e.g., MEG or EEG), functional connectivity usually refers to statistical dependencies between time series of electromagnetic signals from a pair of brain regions. Functional connectivity is considered to arise from the synchronization of presynaptic potentials in a set of neurons in one brain region, which enhances their effect on postsynaptic neurons in the other.

We used the PLI to estimate functional connectivity between the signal sources (18). Although the functional interactions between the estimated signal sources can be captured by quantifying the phase relationships between their time series (19), the reconstructed signal sources may contain artificial spurious interactions due to the field spread. In this case, artificial synchronization may be observed due to the instantaneous linear mixing of the activities of nearby signal sources (20). This kind of artificial synchrony disappears by suppressing zero-delay synchronization. The PLI is an example of a mixing-insensitive interaction index that attenuates artificial interactions. We used the PLI to evaluate phase synchronization between the signal source pairs in each frequency band; the PLI value ranges from 0 to 1, and a PLI of 1 indicates exact synchronization. Note that the PLI does not indicate which of two signals is leading in phase [see (18)].

**1.9 Graph metrics**

We adopted the most commonly used indicators in graph theory. In this theory, a network is defined as a set of nodes or vertices and the edges or lines between them (21). The number of connections between the nearest neighbors of a node as a proportion of the maximum number of possible connections, the average of which is expressed as clustering coefficient (*C*), represents how clustered a graph’s nodes are. If the nearest neighbors of a node are also directly connected to each other, they form a cluster. The clustering coefficient quantifies the number of connections that exist between the nearest neighbors of a node as a proportion of the maximum number of possible connections (22). Random networks (i.e., a randomly generated network with the same number of nodes and edges) have low average clustering, whereas complex networks have high clustering, which is associated with high local efficiency of information transfer and robustness (21). C is a measure for the functional segregation of the brain because the presence of clusters in functional networks suggests the organization of segregated neural processing (21). Path length (*L*) is the minimum number of edges that must be traversed from one node to another (21). Random and complex networks have short average path lengths (i.e., high global efficiency of parallel information transfer), whereas regular networks have long average path lengths (21). The length of a path (the number of edges in the path) estimates the likelihood of functional integration between brain regions, and the shorter the path, the greater the likelihood of integration. Moreover, *L* is the average shortest path length between all node pairs in the network, which is the most commonly used measure of functional integration (23). As a result, if the network contains disconnected nodes, the average path length also diverges to infinity. To avoid this difficulty, we calculate only from connected nodes according to a method used in our earlier studies (16,17). The expected number of such disconnected nodes in a 68-node graph, however, is small for κ = 0.2 (24,25).

Human brain networks are known to show both higher functional integration and higher functional segregation (26). The small-world organization is an intermediate between that of random networks (the short overall path length of which is associated with a low level of local clustering) and that of regular networks (the high level of clustering which is accompanied by a long path length) (22). In this context, small-worldness (*SW*), another graph metric, is the ratio of normalized *C* and normalized *L*. As such, a graph with a high *SW* is a network that is significantly more clustered than a random network yet has approximately the same characteristic path length (27). *SW* is an index that reflects this property in a single statistic (28). For each participant, we calculated *C*, *L*, and *SW* on each frequency band.

**1.10 Data and code availability statement**

All data will be made available to the ethics committee of Kanazawa University. Requests to access the datasets should be addressed to the corresponding author.

**2 Results**

2.1 Group differences in network-level graph metrics

Table S1 summarizes the differences in graph metrics among the groups. The p-values are described after adjustment using the false discovery rate method (29). We observed a similar pattern for other κ values. Figure S1 and Table S2 show the relevant results.

2.2 Group differences in node-level graph metrics

We also examined the node-level graph metrics to determine whether the differences in node-level characteristics could explain the differences in network-level characteristics. When κ was set at 0.10, a substantial portion of the nodes was isolated. Hence, we present the results for κ from 0.12 to 0.3 at 0.02 increments (Table S3).

2.3 Linear relation between *SW* and SRS total T-scores

A linear regression model was used to investigate the relationship between *SW* and SRS total T-score. The relevant results are shown in Table S4 and Figure S2.

**3 Figure Legends**

**Figure S1. Group differences in graph metrics for each frequency band**

The panels present the group differences in graph metrics for different proportional thresholds for each frequency band. Means of the respective graph metrics are presented for each group. ASD, children with autism spectrum disorder; *SW*, small-worldness *C*, clustering coefficient; *L*, path lengths.

**Figure S2. Relationship of small-worldness (*SW*) and SRS total T-scores**

The panels present the relationship between the phase lag index values of the *SW* for each frequency band and SRS total T-scores. SRS, Social Responsiveness Scale; *SW*, small-worldness.

4 Supplementary Tables

Supplementary Table 1. Group differences in graph metrics

Supplementary Table 2. Group differences in graph metrics of thresholds 10 to 30

Supplementary Table 3. Group differences in node-level graph metrics

Supplementary Table 4. Relationship of *SW* and SRS total T-scores

**5 References**

1. Constantino JN, Gruber CP. Social Responsiveness Scale: Manual. Los Angeles, CA: Western Psychological Services (2005).
2. Constantino JN, Todd RD. Autistic traits in the general population: a twin study. Arch Gen Psychiatry (2003) 60:524-30. doi: 10.1001/archpsyc.60.5.524
3. Oka T, Ishikawa SI, Saito A, Maruo K, Stickley A, Watanabe N, et al. Changes in self-efficacy in Japanese school-age children with and without high autistic traits after the Universal Unified Prevention Program: a single-group pilot study. Child Adolesc Psychiatry Ment Health (2021) 15:42. doi: 10.1186/s13034-021-00398-y
4. Kikuchi M, Shitamichi K, Yoshimura Y, Ueno S, Remijn GB, Hirosawa T, et al. Lateralized theta wave connectivity and language performance in 2- to 5-year-old children. J Neurosci (2011) 31:14984-8. doi: 10.1523/JNEUROSCI.2785-11.2011
5. Hasegawa C, Ikeda T, Yoshimura Y, Hiraishi H, Takahashi T, Furutani N, et al. Mu rhythm suppression reflects mother-child face-to-face interactions: a pilot study with simultaneous MEG recording. Sci Rep (2016) 6:34977. doi: 10.1038/srep34977
6. Hayashi N, Kikuchi M, Sanada S, Minabe Y, Miyati T, Hachiman Y, et al. Algorithm for estimation of brain structural location from head surface shape in young children. Neuroreport (2012) 23:299-303. doi: 10.1097/WNR.0b013e3283511de0
7. Yoshimura Y, Kikuchi M, Hayashi N, Hiraishi H, Hasegawa C, Takahashi T, et al. Altered human voice processing in the frontal cortex and a developmental language delay in 3- to 5-year-old children with autism spectrum disorder. Sci Rep (2017) 7:17116. doi: 10.1038/s41598-017-17058-x
8. Anderson V, Hiramoto M, Wong A. Prosodic analysis of the interactional particle ne in Japanese gendered speech. Japanese/Korean Linguistics (2007) 15:43-54.
9. Kajikawa S, Amano S, Kondo T. Speech overlap in Japanese mother-child conversations. J Child Lang (2004) 31:215-30.
10. Tadel F, Baillet S, Mosher JC, Pantazis D, Leahy RM. Brainstorm: a user-friendly application for MEG/EEG analysis. Comput Intell Neurosci (2011) 2011:879716. doi: 10.1155/2011/879716
11. Pernet C, Garrido MI, Gramfort A, Maurits N, Michel CM, Pang E, et al. Issues and recommendations from the OHBM COBIDAS MEEG committee for reproducible EEG and MEG research. Nat Neurosci (2020) 23:1473-83. doi: 10.1038/s41593-020-00709-0
12. Dale AM, Liu AK, Fischl BR, Buckner RL, Belliveau JW, Lewine JD, et al. Dynamic statistical parametric mapping: combining fMRI and MEG for high-resolution imaging of cortical activity. Neuron (2000) 26:55-67. doi: 10.1016/s0896-6273(00)81138-1
13. Huang MX, Mosher JC, Leahy RM. A sensor-weighted overlapping-sphere head model and exhaustive head model comparison for MEG. Phys Med Biol (1999) 44:423-40. doi: 10.1088/0031-9155/44/2/010
14. Baillet S, Mosher JC, Leahy RM. Electromagnetic brain mapping. IEEE Signal Process Mag (2001) 18:14-30.
15. Desikan RS, Ségonne F, Fischl B, Quinn BT, Dickerson BC, Blacker D, et al. An automated labeling system for subdividing the human cerebral cortex on MRI scans into gyral based regions of interest. Neuroimage (2006) 31:968-80. doi: 10.1016/j.neuroimage.2006.01.021
16. Hirosawa T, An KM, Soma D, Shiota Y, Sano M, Kameya M, et al. Epileptiform discharges relate to altered functional brain networks in autism spectrum disorders. Brain Commun (2021) 3:fcab184. doi: 10.1093/braincomms/fcab184
17. Soma D, Hirosawa T, Hasegawa C, An KM, Kameya M, Hino S, et al. Atypical Resting State Functional Neural Network in Children With Autism Spectrum Disorder: Graph Theory Approach. Front Psychiatry (2021) 12:790234. doi: 10.3389/fpsyt.2021.790234
18. Stam CJ, Nolte G, Daffertshofer A. Phase lag index: assessment of functional connectivity from multi channel EEG and MEG with diminished bias from common sources. Hum Brain Mapp (2007) 28:1178-93. doi: 10.1002/hbm.20346
19. Pereda E, Quiroga RQ, Bhattacharya J. Nonlinear multivariate analysis of neurophysiological signals. Prog Neurobiol (2005) 77:1-37. doi: 10.1016/j.pneurobio.2005.10.003
20. Palva S, Palva JM. Discovering oscillatory interaction networks with M/EEG: challenges and breakthroughs. Trends Cogn Sci (2012) 16:219-30. doi: 10.1016/j.tics.2012.02.004
21. Bullmore E, Sporns O. Complex brain networks: graph theoretical analysis of structural and functional systems. Nat Rev Neurosci (2009) 10:186-98. doi: 10.1038/nrn2575
22. Watts D, Strogatz S. Collective dynamics of ‘small-world’ networks. Nature (1998) 393:440-2. doi: 10.1038/30918
23. Rubinov M, Sporns O. Complex network measures of brain connectivity: uses and interpretations. Neuroimage (2010) 52:1059-69. doi: 10.1016/j.neuroimage.2009.10.003
24. Bollobás B, Thomason AG. Threshold functions. Combinatorica (1987) 7:35-8.
25. Graham AJ, Pike DA. A Note on thresholds and connectivity in random directed graphs. Atl Electron J Math (2008) 3:1-5.
26. Tononi G, Sporns O, Edelman GM. A measure for brain complexity: relating functional segregation and integration in the nervous system. Proc Natl Acad Sci U S A (1994) 91:5033-7. doi:10.1073/pnas.91.11.5033
27. Sporns O, Zwi JD. The small world of the cerebral cortex. Neuroinformatics (2004) 2:145-62. doi: 10.1385/NI:2:2:145
28. Bassett DS, Bullmore E. Small-world brain networks. Neuroscientist (2006) 12:512-23. doi: 10.1177/1073858406293182
29. Benjamini Y, Hochberg Y. On the adaptive control of the false discovery rate in multiple testing with independent statistics. J Educ Behav Stat (2000) 25:60-83. doi: 10.1177/1073858406293182
