# Supplementary figures and images for: Alterations in brain networks in children with sub-threshold autism spectrum disorder: A magnetoencephalography study
Source: Front Psychiatry. 2022 Aug 5;13:959763. doi: 10.3389/fpsyt.2022.959763 (PMC9390481; doi:10.3389/fpsyt.2022.959763)

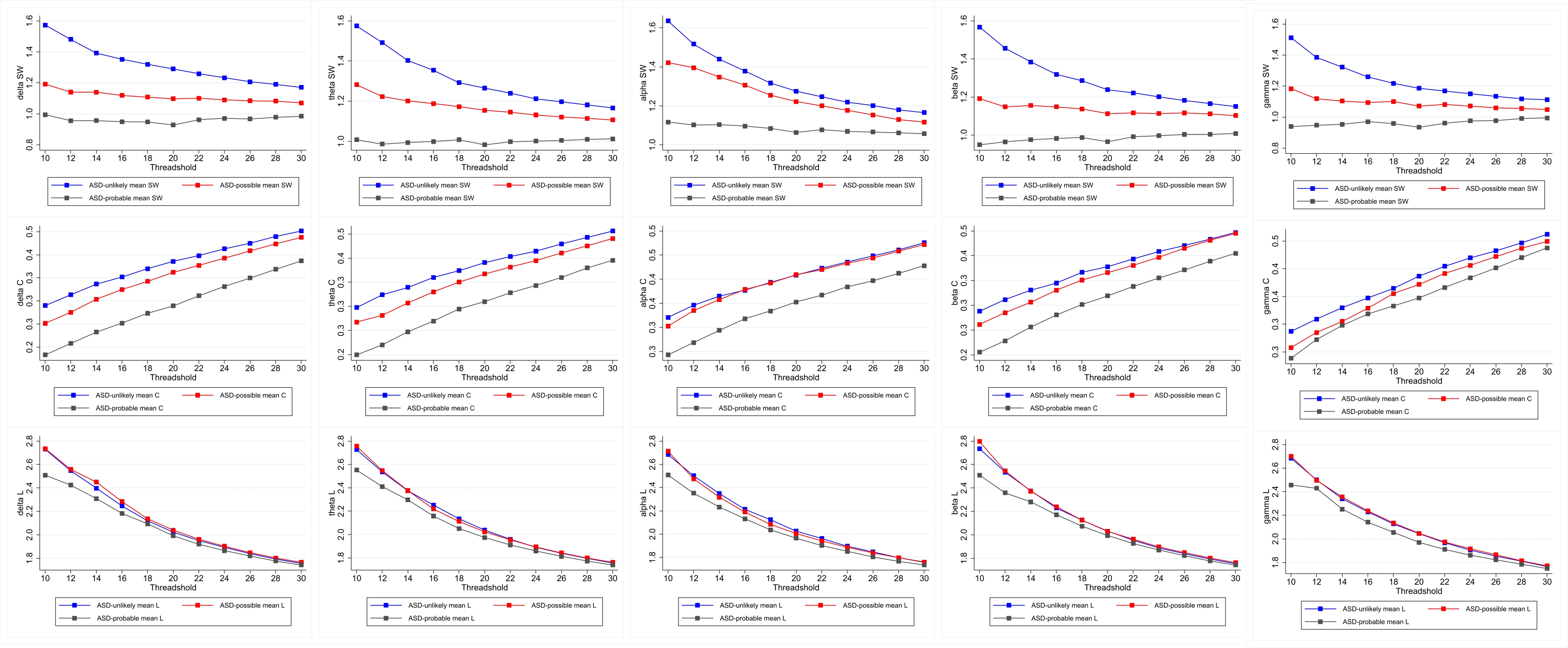

Supplement: Supplementary file 3 [file Image_1.tif]

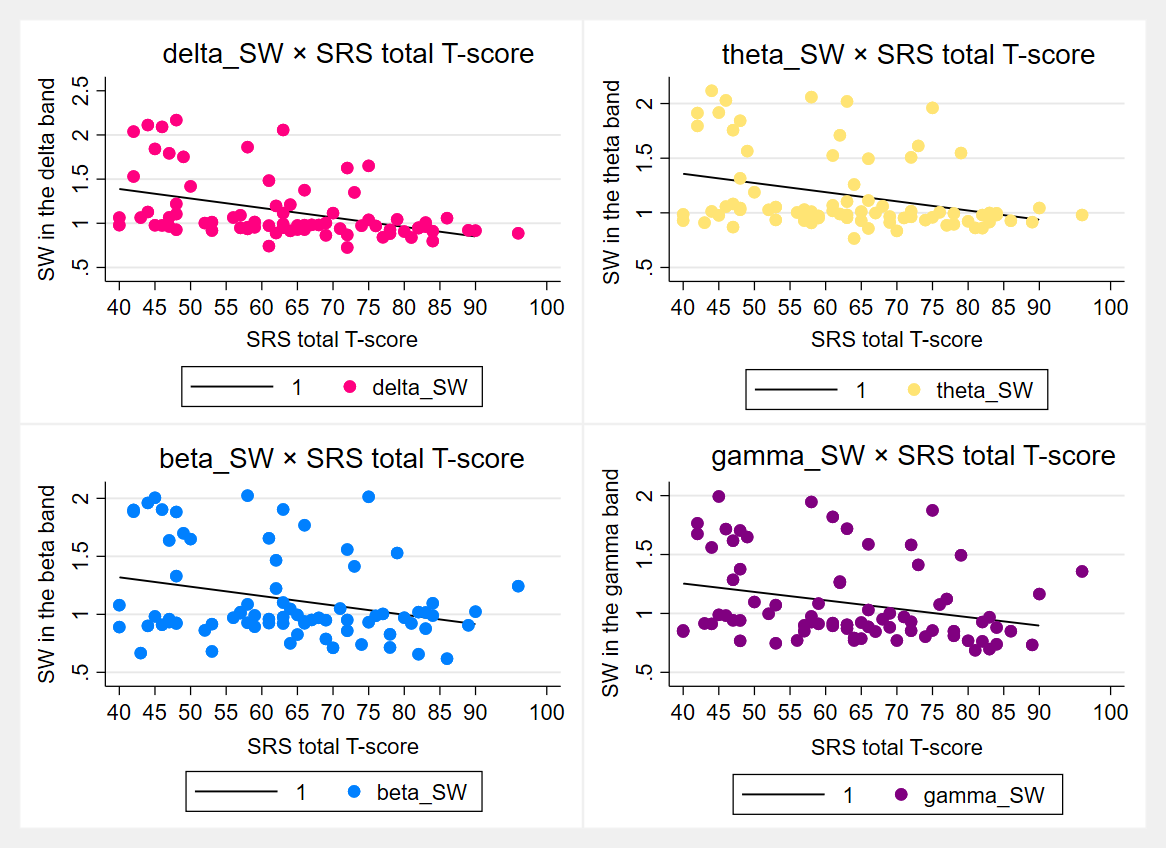

Supplement: Supplementary file 4 [file Image_2.tif]
